# Multifocal Osteomyelitis in an Adolescent Patient With Cat Scratch Disease

**DOI:** 10.1155/2024/9562634

**Published:** 2024-09-07

**Authors:** Burcu Topçu, Hale Usluer Gönüllü, Osman Yeşilbaş, Pınar Polat Suma, Ahmet Soysal

**Affiliations:** ^1^ Division of Pediatrics Ataşehir Memorial Hospital, İstanbul, Türkiye; ^2^ Division of Pediatric Critical Care Medicine Ataşehir Memorial Hospital, İstanbul, Türkiye; ^3^ Division of Radiology Ataşehir Memorial Hospital, İstanbul, Türkiye; ^4^ Division of Pediatric Infectious Disease Ataşehir Memorial Hospital, İstanbul, Türkiye

**Keywords:** *Bartonella henselae*, cat scratch disease, magnetic resonance imaging, osteomyelitis

## Abstract

Cat scratch disease (CSD) typically presents as a self-limiting lymphadenopathy associated with a cat bite or scratch and commonly affects children and young adults. Atypical manifestations, involving the eyes, nervous system, heart, liver, spleen, skin, and musculoskeletal system, could be severe and difficult to diagnose, and they could occur in 5%–20% of the cases. Herein, we report an unusual case of CSD with multifocal osteomyelitis. A 15-year-old girl presented with right axillary lymphadenopathy. Since she had a history of cat scratch, azithromycin was started for CSD. Two days later, she was readmitted to our hospital with severe back pain which required differential diagnosis. Lumbar spinal magnetic resonance imaging (MRI) revealed multifocal vertebral osteomyelitis. The patient was hospitalized, and then teicoplanin and cefotaxime were added to the azithromycin therapy. After excluding the possible other causes, the definitive diagnosis of osteomyelitis secondary to CSD was made upon the combination of the MRI scan findings of the vertebrae, histopathological investigation of excisional right axillary biopsy, positron-emission tomography/computed tomography (PET/CT), and *Bartonella henselae* serologies. Atypical manifestations of CSD are widely variable; therefore, it should be kept in mind in the differential diagnosis of severe musculoskeletal pain and osteomyelitis.

## 1. Introduction

Cat scratch disease (CSD) is a ubiquitous zoonotic infection caused by *Bartonella henselae* and usually affects children and young adults. It mostly develops following particularly a scratch or a bite by a cat, especially a kitten. The most common clinical manifestations (∼90%) in immunocompetent individuals are solitary subacute regional lymphadenopathy associated with systemic symptoms, such as fever, night sweats, and fatigue. In 85% of typical CSD patients, a single lymph node is affected. The axillary and epitrochlear lymph nodes are most frequently affected [1, 2].

Bone infection secondary to CSD is extremely uncommon, and the diagnosis can be missed [2]. In this report, we presented a 15-year-old girl diagnosed with multifocal osteomyelitis secondary to CSD.

## 2. Case Presentation

**Figure 1:**
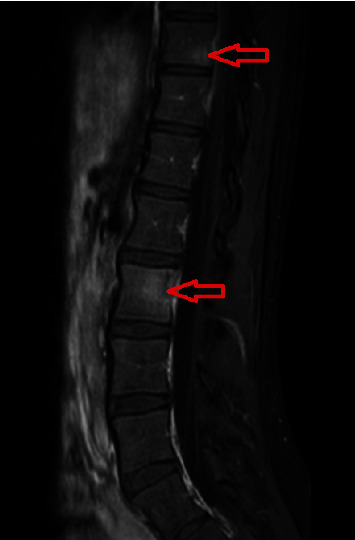
Increased signal intensity at the inferior endplate of the T10 vertebral body and at the posterior region of the L2 vertebral body (red arrows) in the contrast-enhanced T1-weighted sagittal lumbar spinal magnetic resonance imaging scan.

**Figure 2:**
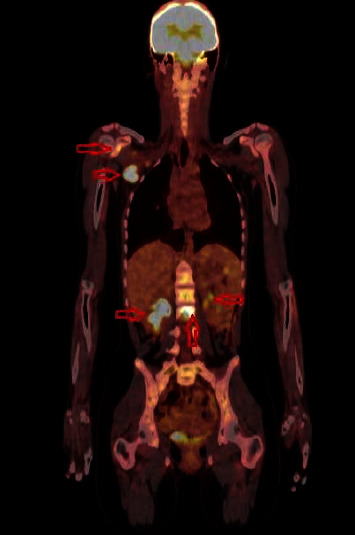
Positron-emission tomography/computed tomography: hypermetabolic involvement in the neck of the right scapula, T10 L2 vertebrae, hypermetabolic conglomerated lymphadenopathies in the right axillary region, hypodense hypermetabolic regions in the liver anterior of segments 5-6, and increased focal activity in the splenic hilus (red arrows).

A 15-year-old girl with no previous complaint was admitted to our pediatrics outpatient clinic with swelling in the right axillary region. She had been describing high fever and low back pain only in the evenings for four days and axillary pain and swelling for a week. Her physical examination was normal except for 20 × 10 mm in diameter of lymphadenopathy in the right axillary region. Her laboratory results were as follows: hemoglobin 11.2 g/dL, white blood cell 8.800/mm^3^, platelet count 188.000/mm^3^, CRP 88.5 mg/L (normal < 5 mg/L), erythrocyte sedimentation rate 64 mm/hour (normal: 0–20 mm/hour), procalcitonin 0.21 ng/mL (normal: < 0.05 ng/mL), aspartate aminotransferase (AST): 65 U/L (normal: 18–36 U/L), and creatinine kinase 1299 U/L (normal: 0–154 U/L). *Brucella* Rose Bengal test, *Brucella* tube agglutination test, Gruber–Widal test, CMV IgM index, *Brucella abortus* IgM, and Quantiferon assay tests were negative. No blasts and atypical cells were observed in the blood smear differential. The other biochemical and electrolyte analyses were unremarkable. Since she had a history of cat scratching, oral azithromycin was prescribed. Two days later, she was readmitted to the hospital with severe back pain. She had painful lumbar flexion, limited range of motion, and paravertebral muscle spasm prominent on the left back side. The straight leg raising test was 50 degrees bilaterally, and no neurological deficit was found. Increased signal intensity at the inferior endplate of the T10 vertebral body and at the posterior region of the L2 vertebral body as consistent with osteomyelitis was detected in the contrast-enhanced T1-weighted sagittal lumbar spinal magnetic resonance imaging (MRI) scan (Figure 1). Her chest x-ray and abdominal ultrasonography (USG) were found to be normal. Teicoplanin and cefotaxime were added to the treatment since the MRI scan findings and possible diagnosis of osteomyelitis. Her axillary USG examination revealed five pathological featured lymph nodes suggesting an infectious or malignant process. Her positron-emission tomography/computed tomography (PET/CT) revealed hypermetabolic involvement in the T10, L2, and neck of the right scapula, hypermetabolic conglomerated lymphadenopathies in the right axillary region, hypodense hypermetabolic regions in the liver anterior of segments five-six, increased focal activity in the splenic hilus, and multiple lymphadenopathies in the peripancreatic and cardia regions (Figure 2). There was no growth in the repeated blood cultures.

In order to exclude malignancy and granulomatous infection, an excisional biopsy was also performed on the lymph nodes in the right axillary region. Histopathologic investigation of those lymph nodes showed granulomatous inflammation of the epithelioid histiocytes that have central necrotic regions with microabscesses and also a few Langerhans-type giant cells. The immunohistochemical staining of the specimens' results were as follows: CD68 was positive in the epithelioid histiocytes, CD123 was positive in the focal plasmacytoid dendritic cells, myeloperoxidase was positive in the leukocytes in the abscess region, CD20 was positive in the B lymphocytes around necrosis, and CD3 was positive in the T lymphocytes around necrosis. All of these findings were consistent with CSD. The acid-fast *bacillus* test was negative in the acid-fast bacteria staining. Chronic granulomatous disease was excluded since the dihydrorhodamine test was negative. A commercial ELISA serological method was used to confirm the diagnosis of CSD. The diagnosis was based on elevated titers of IgM (>100) or IgG (>320). *B. henselae* IgM and IgG were + and ++ positive, respectively. As a result of this, rifampicin was added to her treatment. Cefotaxime and azithromycin were continued with routine analgesia because of her persistent and severe back pain. Her eye examination was normal. After 4 weeks of hospitalization, the patient was discharged with oral ciprofloxacin, azithromycin, and rifampicin. After 3 weeks of the discharging, the lumbar MRI still showed contrast uptake in the T10 vertebra's left inferior end region and posterior corpus of the L2 vertebra. The patient's treatment was completed in 8 weeks. The patient was followed up by the orthopedics clinic for 18 months, and she has fully recovered from multifocal osteomyelitis secondary to CSD.

## 3. Discussion

Atypical manifestations of CSD can affect the nervous system, heart, eyes, liver, skin, spleen, or musculoskeletal system and that can be severe and difficult to diagnose. These involvements may occur in 5%–20% of patients with CSD. Osteomyelitis and other musculoskeletal involvements are considered to be exceedingly rare in CSD [2, 3]. In a surveillance study conducted over 11 years, Maman et al. [2] showed that 96 patients (10.5%) had musculoskeletal manifestations in 913 patients in total with CSD. In this study, it was revealed that myalgia occurred in 53 patients (5.8%) and was often severe, with a median duration of 4 weeks. Arthralgia and/or arthritis occurred in 50 patients (5.5%) mainly in the medium and large joints with a median duration of 5.5 weeks. Tendinitis, neuralgia, and osteomyelitis were observed in seven, four, and two patients, respectively. One of the remarkable conclusions of this study is that the patients with musculoskeletal manifestations were significantly older than patients in the control group [2]. Our patient was diagnosed with multifocal osteomyelitis secondary to CSD at the age of 15, which is older than the disease's peak incidence between 2 and 14 years old [1].

It is stated that osteomyelitis is a well-known yet very rare atypical manifestation of CSD accounting for 0.17%–0.27% [3, 4]. In their literature reviews, Donà et al. [4] and Hajjaji et al. [5] have analyzed 52 and 47 patients with osteomyelitis secondary to CSD, respectively. Sex distribution was reported as equal in both of the two reviews. The average ages were 7, 8, and 9 years, respectively. It was revealed that vertebral bodies followed by limbs were the most affected bones during CSD [4, 5]. Fever and bone pain were the main symptoms [5]. A solitary bone lesion was the first presentation, and it has been found that multiorgan involvement secondary to CSD was not correlated with multifocal osteomyelitis [4]. Diagnosis is based on serology, histologic analysis of lymph nodes and/or bone biopsy, and PCR tests, sometimes in combination. The skin test is no longer used for the diagnosis of CSD [6]. The differential diagnoses of osteomyelitis secondary to CSD include chronic recurrent multifocal osteomyelitis, bacterial osteomyelitis, chronic granulomatous diseases, malignancy, eosinophilic granuloma, and histiocytosis. It is hypothesized that osteomyelitis secondary to CSD mostly results via hematogenous or lymphatic spread since it frequently affects a bone at a distance from the inoculation area or inflamed lymph node [6]. Our 15-year-old patient was admitted with right axillary lymphadenopathy, and 2 days later, she was readmitted to the hospital with severe back pain prominent on the left side despite appropriate CSD treatment. After excluding the possible other causes, the definitive diagnosis of osteomyelitis secondary to CSD was made upon the combination of the MRI scan findings of the vertebrae, histopathological investigation of excisional right axillary biopsy, PET/CT, and *B. henselae* serologies.

Uncomplicated CSD does not require any antibiotic therapy since it is thought that the clinical manifestations of the disease, especially lymphadenopathy, are relevant to hyperactivation of immunoinflammatory pathways. There are many publications reporting various antimicrobial regimens for CSD [7, 8]. However, Bass et al. [9] have conducted the only prospective, double-blind, placebo-controlled treatment trial with azithromycin in immunocompetent patients with uncomplicated CSD. In this study, oral azithromycin treatment for 5 days has demonstrated clinical efficacy as measured by a total decrease in lymph node volume [9]. Although our patient had been treated with azithromycin since her first admission, the treatment has failed to prevent CSD progression to multifocal osteomyelitis. The efficacy of antibiotics in the treatment of osteomyelitis secondary to CSD is mainly obtained from retrospective studies due to its rareness [4]. Since the lack of comparative data between antibiotic regimens, there are no evidence-based guidelines. A literature review by Hajjaji et al. [5] has presented the various antibiotics used sequentially or in combination with different durations for osteomyelitis secondary to CSD that have been associated with full recovery. In our case, we added teicoplanin and cefotaxime on the third day of azithromycin since the MRI scan findings and possible diagnosis of osteomyelitis. After confirming the CSD diagnosis by the serological results, rifampicin was added to her treatment. We continued these antibiotics for 4 weeks during hospitalization. The patient was discharged with oral ciprofloxacin, azithromycin, and rifampicin. We continued these oral antibiotics for 8 weeks, and they were stopped after regression of the bone lesions in the control MRI. No complications such as medullar compression and bone abscesses were seen in our patient.

In conclusion, osteomyelitis is an extremely rare atypical manifestation of CSD and it could be severe and difficult to diagnose. Further studies are warranted to determine the optimal antibiotic regimen for this unusual involvement of CSD.

## Data Availability

The data and materials used to support the findings of the study are available from the corresponding author upon request.
